# Strategies aiming to improve statin therapy adherence in older adults: a systematic review

**DOI:** 10.1186/s12877-024-05031-z

**Published:** 2024-05-21

**Authors:** Philipp Frieden, Rose Gagnon, Élodie Bénard, Benoît Cossette, Frédéric Bergeron, Denis Talbot, Jason Robert Guertin

**Affiliations:** 1 Axe Santé des Populations et Pratiques Optimales en Santé, Centre de recherche du CHU de Québec – Université Laval, Hôpital du Saint-Sacrement, 1050 chemin Sainte-Foy, local J1-11, Quebec City, QC G1S 4L8 Canada; 2 Faculté de médecine, Université Laval, Quebec City, QC Canada; 3 Centre de recherche en organogénèse expérimentale de l’Université Laval/LOEX, Quebec City, QC Canada; 4 Centre interdisciplinaire de recherche en réadaptation et intégration sociale (Cirris), Quebec City, QC Canada; 5 Centre de recherche sur le vieillissement du CIUSSS de l’Estrie – CHUS, Sherbrooke, QC Canada; 6 Faculté de médecine et des sciences de la santé, Université de Sherbrooke, Sherbrooke, QC Canada; 7 Bibliothèque de l’Université Laval, Quebec City, Qc Canada

**Keywords:** Statin, Older adult, Adherence, Systematic review

## Abstract

**Background:**

Randomized clinical trials have shown that, under optimal conditions, statins reduce the risk of cardiovascular events in older adults. Given the prevalence and consequences of suboptimal adherence to statin among older adults, it is essential to document strategies designed to increase statin adherence in this population. The objective of this systematic review is to describe and summarize the effectiveness of interventions to improve statin adherence in older adults (≥ 65 years old).

**Methods:**

This review followed PRISMA guidelines. Studies were identified from PubMed, PsycINFO, Embase, CINAHL and Web of Science. Study selection was conducted independently by four reviewers working in pairs. Included studies reported data on interventions designed to increase adherence to statin therapy in older adults and were original trials or observational studies. Interventions were pragmatically regrouped into 8 different categories going from patient to administrative level. Two reviewers extracted study data and assessed study quality independently. Given the heterogeneity between the included studies, a narrative critique and summary was conducted.

**Results:**

Twelve out of the 2889 identified articles were included in the review. Our review showed that simplifying patients’ drug regimen, administrative improvements and large-scale pharmacy-led automated telephone interventions show positive effects on patient adherence to statin therapy, with odds ratios between > 1.0 and 3.0, while education-based strategies and intensified patient care showed mixed results.

**Conclusions:**

Current evidence suggests that some interventions can increase statin adherence in older adults, which could help in the reduction of the risk of a cardiovascular event in this population.

**Supplementary Information:**

The online version contains supplementary material available at 10.1186/s12877-024-05031-z.

## Background

Cardiovascular disease (CVD) is the leading cause of death worldwide [1] and the second in Canada [2]. The total cost associated with CVD in Canada has been evaluated at $22 billion in 2010 [3], which makes CVD the second most costly illness in the country. Older adults (≥ 65) are particularly vulnerable to CVD and are predicted to make up one quarter of the Canadian population in the next 20 years [4]. Therefore, identifying strategies aimed at preventing CVD in older adults is of capital importance not only for healthy aging, but also to control expenses of the Canadian healthcare system [5].

Several randomized clinical trials (RCTs) have shown that statins reduce the risk of a first cardiovascular event in people aged 65 and older [6–11]. Statin use after a cardiovascular event has also been shown to reduce the mortality rate and the risk of subsequent cardiovascular events in patients [11–13]. Indeed, in 2013, a Cochrane meta-analysis focusing on primary prevention showed a 14% reduction in all-cause mortality, a 27% reduction in coronary heart disease, a 25% reduction in CVD and a 22% reduction in strokes [9] in statin-treated patients in that age category. In addition, RCTs have shown that drugs in this class have little to no severe side effects [9, 11]. Recently, another meta-analysis of RCTs published in 2019 found that patients aged > 65 to 70 years old in primary prevention treated with statins had a 39% reduced risk of having a major cardiovascular event [12].

Of course, the effectiveness of a statin treatment depends, among other factors, on patient adherence to treatment, i.e., “the process by which patients take their medication as prescribed” [13]. Indeed, treatments may show suboptimal results if they are not taken as prescribed. A meta-analysis published in 2017 showed that adherence to statins prescribed for primary or secondary prevention of CVD in patients aged 65 years and older was not optimal [14]. Indeed, after a year of treatment, 60% of older adults were adherent while 23% had ceased taking the medication [14]. Results observed in another meta-analysis with middle-aged and older adults showed that patients with poor adherence to statin therapy had a 15% higher risk of developing CVD than those with optimal adherence and the risk of all-cause mortality in adherent patients was 45% lower (RR = 0.55 [0.46–0.67]) when compared to patients with poor adherence [15].

As such, several groups have sought to develop and implement interventions aimed at increasing statin adherence [16]. Given the beneficial effects of statin therapy in the prevention of CVD in older adults as well as the prevalence and consequences of suboptimal adherence, it is essential to document strategies designed to increase statin treatment adherence in this population. We therefore sought to describe and evaluate the effectiveness of interventions aiming to improve statin adherence in individuals aged 65 years and older. By doing so, we aim to provide a comprehensive assessment of the literature for the design and subsequent evaluation of interventions aimed at improving adherence in older adults.

## Methods

This review is reported following the *Preferred Reporting Items for Systematic Reviews and Meta-Analyses* (PRISMA) 2020 [17] (for completed checklist, see Additional File 1). The review and its protocol were not registered.

### Eligibility criteria

The eligibility criteria were defined following a PICOS (population, intervention, comparators, outcomes, study design) approach [18].

#### Population

The selected studies were conducted at least in part on individuals aged 65 years and older undergoing statin treatment in a context of prevention of CVD.

#### Intervention

The studied intervention could be any intervention with the explicit goal of increasing the patient’s adherence to statin therapy. Aligned with a past review on a similar subject [19], interventions were split into 8 categories: (1) simplification of drug regimen; (2) patient education and information; (3) intensified patient care (increased follow-up, sending out reminders, etc.), (4) complex behavioural approaches (increasing motivation by arranging group sessions, giving out rewards, etc.); (5) decision support systems (computer‐based information systems aimed at support of decision‐making); (6) administrative improvements (audit, documentation, automatic prescription refill program, co‐payments); (7) large‐scale pharmacy‐led automated telephone interventions; and (8) other interventions.

#### Comparators

Comparison groups had to include patients who were not receiving interventions to increase their adherence to statin therapy or who received what is considered usual care.

#### Outcomes of interest

Adherence to statins, discontinuation or proportion of dispensation had to be a primary or secondary goal of the intervention studied in the selected articles.

#### Study design

Studies could be randomized and non-randomized clinical trials, controlled before-after studies, as well as studies with repeated data measures and discontinued time-series studies [20].

### Information sources

We carried out an electronic search on several databases up to January 17th, 2023 without restriction on the date of publication of the studies or on the language of writing. The databases used were PubMed, Embase, PsycInfo, Web of Science and CINAHL.

### Search strategy

The search strategy (Additional File 2) was initially developed for PubMed by consulting the literature [21, 22], with the collaboration of a research librarian (FB). The search strategy uses a mix of free and controlled vocabulary (i.e. MeSH) related to the concepts of outcomes (adherence to treatment), medication (statins) and the population under study (65 years and older). The Boolean operator AND was used to limit the search to these three concepts. The strategy was also translated for Embase (Embase.com), PsycInfo (Ovid), Web of Science and CINAHL.

### Selection process

Four individuals were part of the selection process (PF, RG, EB, JRG). All four authors screened half of the records. As a result, each record was screened once by two of the four authors, with another one intervening in case of discordant rulings. In order to maximize consistency between authors, the first 100 citations were screened by all four reviewers and discrepancies were discussed amongst them prior to concluding the screening process.

The selection of the articles was realized in four steps:

### Identification of the reviewers

The four reviewers (PF, RG, EB, JRG) reviewed the articles found in the selection process.

### Selection of the articles by title and abstract

Articles found by the electronic search were compiled into Covidence, a screening and data extraction tool used for systematic reviews [23]. The importation process automatically eliminated duplicates. Each of the reviewers used Covidence to select articles by examining their titles and abstracts. To be included in the next selection phase, the article had to be selected by two reviewers independently. Discordant results were then addressed following a discussion and consensus of the reviewers. If the abstract was missing, it was added manually. If it was not possible to find an abstract or a full text, the article was excluded. If an article was found to be in a language none of the four authors were fluent in (English, French, German) and no individual capable of translating it could be found, it was not included.

### Selection of the articles by full text

The full texts of selected articles were uploaded to Covidence in PDF format if they were not already present. The reviewers read the articles independently. The selection process was identical to the last phase.

### Data collection process

Data was collected by all reviewers using a Covidence data extraction form.

### Data items

Outcome data was sought for measures of adherence to statin treatment, measured in percentage of days covered (PDC), medication possession ratio (MPR) or self-reported adherence. All results that were compatible with the studied outcome were sought when the outcome was measured on individuals aged 65 years and older.

Other considered variables were: study characteristics, including the family name of the first author, the year of publication, the study design, the country in which the study was conducted, the participation rate, the population characteristics, including the initial sample size, the intervention and control group sizes, the mean age/range in years, the number of women included in the study, the population from which the sample of the study’s participants is drawn and the setting of the study, the participants follow-up, including the type of statin prescribed, the strategies used to keep the participants until the end of the study, the duration of the participants follow-up, the number and percentage of participants at the end of the study in total and in each group, the characteristics of interventions, including the ones offered to the intervention and the control groups, and the characteristics of outcomes, including the type of outcome measures and the effectiveness of the interventions. Missing or unclear information was clarified by contacting the authors.

### Study risk bias assessment

Risk of bias in the included studies were assessed with the Joanna Briggs Institute’s (JBI) critical appraisal tools [24] by PF and RG.

### Effect measures

Adherence to statin treatment is presented in odds ratios, risk ratios and hazard ratios and by a standardized mean difference of the adherence measure between intervention and control groups.

### Statistical analysis

Each intervention was assigned to one of the eight intervention categories outlined in the eligibility criteria; in cases where the intervention combined multiple components, a single dominant component was determined by consensus amongst reviewers involved in the data extraction.

Study results pertaining to statin adherence were presented as they were shown in the articles. If essential summary statistics were missing, we contacted the authors to obtain them.

The characteristics of the included studies and syntheses are presented by intervention category and type of adherence measure. Due to the heterogeneity between intervention types, it was deemed inappropriate to pool estimates of the different studies together. For the same reason, we considered that a meta-analysis would not be appropriate. Therefore, the results were synthesized in a narrative manner and sensitivity analyses to assess the robustness of the results were not conducted.

### Reporting bias and certainty assessment

The assessment of reporting bias and certainty was not applicable in the context of this systematic review as no meta-analysis was done due to the heterogeneity of the studies’ interventions.

## Results

### Study selection

**Fig. 1:**
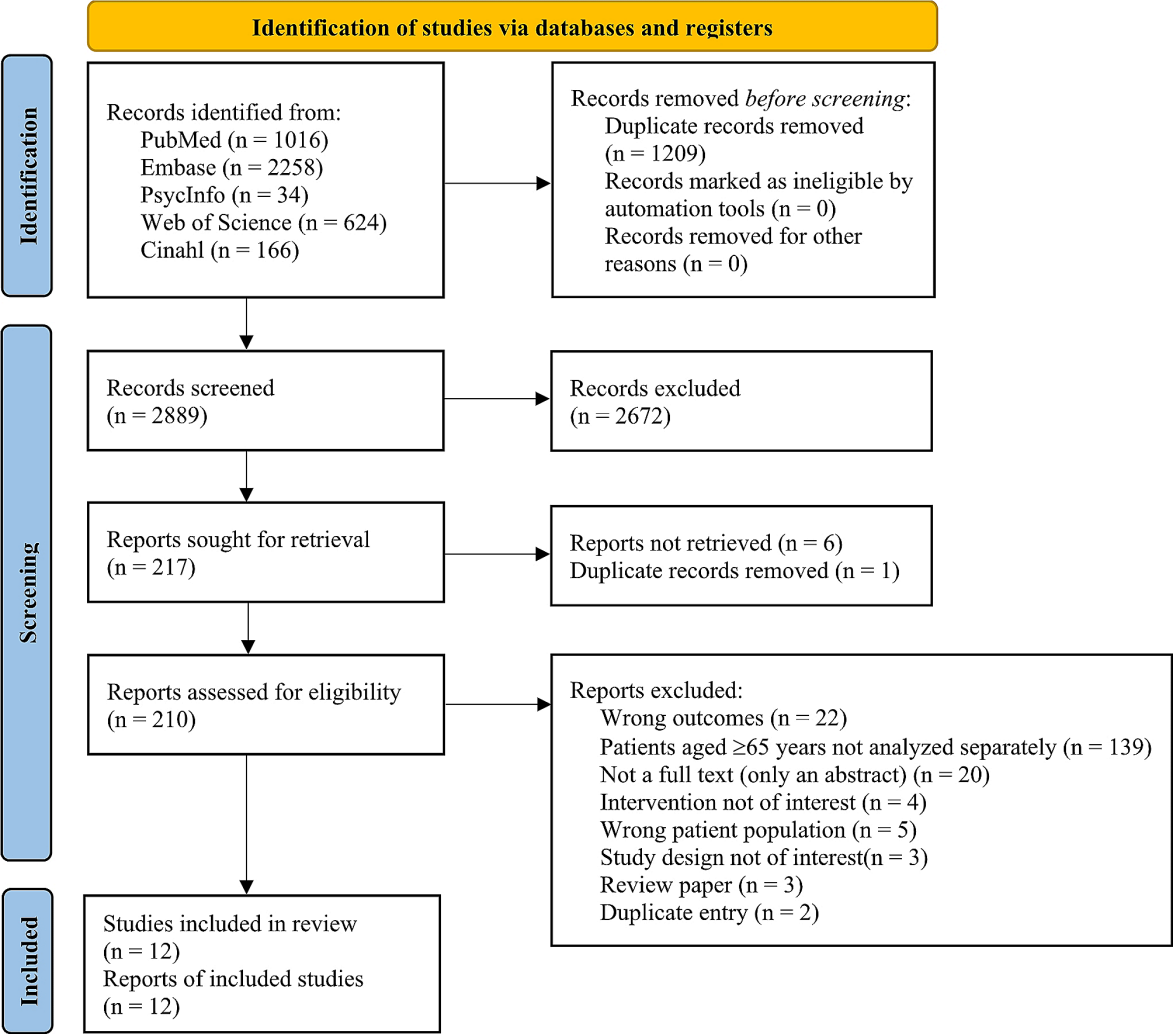
Flowchart for the study selection process

A total of 4098 articles were imported into Covidence (Fig. 1). After removal of 1 209 duplicates, a total of 2 889 articles were retained for selection by title and abstract.

### Study characteristics

Table 1 shows the characteristics of the studies included in this review, with studies being grouped by intervention type, and by adherence measure. Out of 12 included studies, six had older adults as the population of interest [25–30], one study included only male patients who were 65 to 74 years old [31], and five included patients 65 years and older as sub-populations [32–36]. Only three studies [25, 27, 30] included patient retention rates for older adults. Outcome measures varied between selected studies (Table 2). They were measured as PDC in nine studies [25–32, 35], in terms of the MPR in one study [34], as discontinuation of statin treatment in two studies [32, 33], and as a proportion of dispensation in one study [36]. More information on intervention characteristics is provided in Additional file 3. All studies were conducted in high-income countries.

**Table 1:** Characteristics of studies included in the analysis

Author/year	Country	Funding source	Setting	Design	N	Sample
Simplification of drug regimen						
Rea et al. (2021)	Italy	Università degli Studi di Milano – Bicocca, Italian Ministry of Education, University and Research, Italian Ministry of Health	Healthcare utilization databases of Lombardy	Cohort study	65-80 y.o.: 1507	Adults aged 40–80 y.o. and beneficiaries of the NHS
Patient education and information						
Eussen et al. (2010)	Netherlands	National Institute for Public Health and the Environment	Community pharmacy	Randomised controlled trial	314	New ≥ 18 y.o. statin users
Qvist et al. (2020)	Denmark	Central Denmark Region Health Science Research Fund, Family Hede Nielsen Fund, Frimodt-Heineke Fund	Central Denmark Region	Randomised controlled trial	435	Males aged between 65–74 y.o. in the screening arm of the VIVA trial diagnosed with AAA or PAD without prior statin or antithrombotic treatment
Intensified patient care						
Casula et al. (2015)	Italy	Regione Lombardia	General practices	Quasi-experimental study	5323	Pre-intervention: patients with 1st statin prescription between January 1st, 2007 and June 30th, 2007 Post-intervention: patients with 1st statin prescription between July 1st, 2008 and December 31st, 2008
Faridi et al. (2016)	United States	Agency for Healthcare Research and Quality	ACTION Registry-GWTG (US quality improvement registry)	Cohort study	20,219	Patients with at least 90 days of prescription coverage from Medicare Part D prior to discharge
Guerard et al. (2018)	United States	Unclear	Medicare Advantage plan diabetic population (administrative database)	Cohort study	≥ 65 years old: 163,628	Medicare Advantage members who received a new diabetes diagnosis.
Kooy et al. (2013)	Netherlands	Utrecht University	Community pharmacies	Randomised controlled trial	381	Patients who received a statin prescription in the preceding month, with a 50–80% yearly refill adherence over the last 12–18 months
Administrative improvements						
Ivers et al. (2013)	Canada	Cardiac Care Network of Ontario, Canadian Institutes for Health Research	Institute for Clinical Evaluative Sciences (population-based administrative records linked through a unique identifier)	Cohort study	16,134	Patients ≥ 65 y.o. with confirmed CAD
Lester et al. (2016)	United States	NIH National Center for Advancing Translational Sciences	29-store pharmacy chain	Quasi-experimental study	4946	Patients aged ≥ 65 y.o. taking ≥ 1 medication in the therapeutic categories used for calculating the PDC measures
Schmittdiel et al. (2015)	United States	Kaiser Permanente Center for Effectiveness and Safety Research, Agency for Healthcare Research and Quality	Health care delivery systems sites	Cohort study	93,276	Diabetic patients aged ≥ 65 y.o. as of January 1st, 2010
Large-scale pharmacy-led automated telephone intervention						
Derose et al. (2013)	United States	Merck Sharp & Dohme Corp	Health plan pharmacy	Randomised controlled trial	≥ 75years old: 2983	Patients ≥ 24 y.o. with a statin prescription and no record of a statin prescription in the year before the index prescription date enrolled with a health plan pharmacy for ≥ 1 year
Vollmer et al. (2014)	United States	Agency for Healthcare Research and Quality	Kaiser Permanente health plan	Randomised controlled trial	≥ 71 years old: 4357	Patients ≥ 40 y.o. with documented diabetes or CVD and ≥ 1 dispensation of ACEI, ARB, or statin in the preceding 12 months, with suboptimal adherence in the preceding 12 months, and were due or overdue for a refill
**Author/year**	**Intervention**	**Control**	**Duration**	**Retention rate**		
Simplification of drug regimen						
Rea et al. (2021)	Statin and ezetimibe prescribed in a single-pill formulation	Statin and ezetimibe prescribed separately	12 months	NR for participants over 65		
Patient education and information						
Eussen et al. (2010)	Pharmacy visits for 5 individual counseling sessions, each lasting 10–15 min	Usual care	12 months	NR for participants over 65		
Qvist et al. (2020)	Telephone-based interview and counselling performed by a study nurse, based on a pragmatically designed semi-structured questionnaire.	Usual care	60 months	NR for participants over 65		
Intensified patient care						
Casula et al. (2015)	Informative and educational intervention addressed to general practitioners	Usual care	12 months	NR for participants over 65		
Faridi et al. (2016)	Earlier first outpatient visit after discharge for STEMI or NSTEMI	Usual care	3 & 12 months	90 days: 89.8% 1 year: 57.4%		
Guerard et al. (2018)	Receiving a CWA in the preceding 12 months of the study	Usual care	60 months	NR for participants over 65		
Kooy et al. (2013)	10-minute pharmacist counseling about non-adherence and a compliance card that signals every 24 h	Usual care	12 months	88.22%		
Administrative improvements						
Ivers et al. (2013)	Increase in the number of days supplied in the initial prescription fill for each medication class	Usual care	18 months	NR for participants over 65		
Lester et al. (2016)	Enrollment in an automatic prescription refill program	Usual care	At least 90 days − 2 months (at least 2 prescription refills)	NR for participants over 65		
Schmittdiel et al. (2015)	Mean days supply of drugs in therapeutic category in 2010, Percentage of drugs in therapeutic category refilled through mail order pharmacy in 2010, Generic drug copayment for 30-day supply in 2010, Annual individual out-of-pocket maximum in January 2010	Usual care	12 months	96.51%		
Large-scale pharmacy-led automated telephone intervention						
Derose et al. (2013)	Providing patients with educational information and an encouraging prompt to adhere to a recently prescribed statin	Usual care	32–39 days	NR for participants over 65		
Vollmer et al. (2014)	IVR calls with or without a personalized reminder letter if patients are due or overdue for a statin refill	Usual care	12 months	NR for participants over 65		

AAA = Abdominal Aortic Aneurysm, ACEI = Angiotensin-converting enzyme inhibitors, ARB = Angiotensin II receptor blockers, CAD = Coronary Artery Disease, CVD = cardiovascular disease, CWA = Comprehensive Wellness Assessment, IVR = Interactive voice recognition, IVR + = Interactive voice recognition with a personalized reminder letter MPR = Medication possession ratio, NHS = National Health Service, NR = Not reported, NSTEMI = Non-ST-elevation myocardial infarction, PAD = Peripheral Artery Disease, PDC = Proportion of days covered, STEMI = ST-elevation myocardial infarction, y.o. = years old

**Table 2:** Outcomes of studies included in the analysis

Author/year	Adherence measure	Outcome measure	Results		Adjustment			
					Sex		Age	Other^†^
Simplification of drug regimen								
Rea et al. (2021)	Proportion of days covered	RR (95% CI)	Association between PDC > 75% and single-pill combination of statin and ezetimibe vs. two-pill or separate administration: 2.12 (1.89–2.38)		X		X	X
	Treatment discontinuation	RR (95% CI)	Association between single-pill vs. two-pill administration of a statin and ezetimibe and treatment discontinuation: 0.68 (0.63–0.74)					
Patient education and information								
Eussen et al. (2010)	Incidence of discontinuation	HR (95% CI)	> 65 years old: 0.903 (0.569–1.433)					
Qvist et al. (2020)	Proportion of days covered	% (95% CI)	6 months	Continuous: Intervention: 78.3%, Control: 71.4%, (*p* = 0.04)				
				PDC ≥ 80%: Intervention: 63.2 (56.8–69.2) Control: 53.4 (46.5–60.2)				
			12 months	Continuous: Intervention: 75.1%, Control: 69.1%, (*p* = 0.12)				
				PDC ≥ 80%: Intervention: 70.1 (63.9–75.7) Control: 63.2 (56.4–69.6)				
			60 months	Continuous: Intervention: 69.2%, Control: 65.7%, (*p* = 0.34)				
				PDC ≥ 80%: Intervention: 57.6 (51.1–63.8) Control: 53.9 (47.0-60.7)				
		PD (95% CI)	6 months	Crude: 9.8 (0.5–19.0), *p* = 0.04				X
				Adjusted: 10.1 (0.9–19.4), *p* = 0.03				
			12 months	Crude: 6.9 (-2.0-15.8), *p* = 0.13				
				Adjusted: 7.1 (-1.7-16.0), *p* = 0.11				
			60 months	Crude: 3.7 (-5.7-13.0), *p* = 0.44				
				Adjusted: 4.0 (-5.3-13.3), *p* = 0.4				
Intensified patient care								
Casula et al. (2015)	Medication possession ratio	Difference (%)	65–79 years old: +6.3 (NR) post-intervention vs. pre-intervention					
			≥ 80 years old: +8.3 (NR) post-intervention vs. pre-intervention					
Faridi et al. (2016)	Proportion of days covered	OR (95% CI) of PDC ≥ 80%	90 days	≤ 1 week: Reference	X		X	X
				1-2 weeks: 0.95 (0.86‐1.04), *p* = 0.25				
				2-6 weeks: 0.98 (0.89‐1.070), *p* = 0.61				
				> 6 weeks: 0.78 (0.70-0.87), *p* < 0.001				
			1 year	≤ 1 week: Reference				
				1-2 weeks: 0.93 (0.83‐1.05), *p* = 0.25				
				2-6 weeks: 0.96 (0.86‐1.07), *p* = 0.43				
				> 6 weeks: 0.78 (0.68-0.89), *p* < 0.001				
Guerard et al. (2018)	Proportion of days covered	IRR	65–80 years old: 1.013 (not significant)		X		X	X
Kooy et al. (2013)	Proportion of days covered	OR (95% CI) of PDC ≥ 80%	Card + Counseling	Crude: 1.22 (0.72–2.06), *p* = 0.45				X
				Adjusted: 1.18 (0.69–2.01), *p* = 0.55				
			Card only	Crude: 1.33 (0.76–2.32), *p* = 0.55				
				Adjusted: 1.49 (0.83–2.69), *p* = 0.18				
			Card only, Secondary prevention in women	Adjusted: 8.26 (2.20–31.0), *p* = 0.002				
Administrative improvements								
Ivers et al. (2013)	Proportion of days covered	N (%) of PDC > 80%	< 31 days	8962 (76.1%)	X		X	X
			31–60 days	887 (87.0%)				
			> 60 days	3019 (90.5%)				
		OR (95% CI) of PDC > 80%	< 31 days	1 (Reference)				
			31–60 days	2.0 (1.7–2.4)				
			> 60 days	3.0 (2.6–3.4)				
Lester et al. (2016)	Proportion of days covered	OR (95% CI) of PDC > 80%	1.51 (1.26–1.82)		X^*^		X	X
Schmittdiel et al. (2015)	Proportion of days covered	RR of PDC ≥ 80%	Mean days supply of drugs in therapeutic category	< 31: Reference	X		X	X
				31–60: 1.11, *p* < 0.001				
				61–90: 1.47, *p* < 0.001				
				> 90: 1.61, *p* < 0.001				
			Mail order pharmacy	0%: Reference				
				1-50%: 1.01, *p* > 0.05				
				51-100%: 1.07, *p* < 0.001				
			Generic drug copayment for 30-day supply	> 10$: Reference				
				$0-$10: 1.02, *p* < 0.01				
			Annual individual out-of-pocket maximum	>$2000: Reference				
				$0-$2000: 1.02, *p* < 0.001				
Large-scale pharmacy-led automated telephone intervention								
Derose et al. (2013)	Proportion of dispensation	OR (95% CI)	> 70 years old: 2.32 (1.70–3.18)		X		X	X
Vollmer et al. (2014)	Proportion of days covered	PD (95% CI)	IVR + vs. UC	0.035 (0.012–0.058), *p* = 0.003	X		X	X
			IVR vs. UC	0.029 (0.006–0.051), *p* = 0.013				
			IVR + vs. IVR	0.006 (-0.017-0.029), *p* = 0.606				

RR = Risk Ratio, CI = Confidence interval, PDC = Proportion of days covered, HR = Hazard Ratio, PD = Proportion difference, NR = Not reported, OR = Odds Ratio, IRR = Incidence rate ratio, IVR + = Interactive voice recognition with a personalized reminder letter, UC = Usual care, IVR = Interactive voice recognition

*: This manuscript adjusted for gender and not sex

†: Additional covariates were adjusted for within these manuscripts; Examples include high blood pressure, diabetes and total number of medications taken by the patient

### Risk of bias in studies

Studies were divided by their design when assessing their risk of bias, with Additional file 4 providing the risk of bias for RCTs, cohort studies and quasi-experimental studies. Results show that selected studies could be prone to bias. In the included RCTs, blinding was often an issue as blinding might not have been feasible. Furthermore, only two [33, 36] out of five RCTs had outcome assessors being blinded to treatment assignment. In addition, follow-up was only complete in one study [31] and differences between groups in terms of their follow-up were not always adequately described or analyzed. In the included cohort studies, follow-up was complete or described and explored if incomplete in only two studies [28, 32]. In the included quasi-experimental studies [29, 34], patients included in the comparisons were not always similar across intervention and control groups. Moreover, multiple measurements of the outcome both pre and post intervention were not done, and it was unclear whether follow-up was complete or adequately addressed if incomplete.

### Results of individual studies

Out of 12 included studies, 10 studies reported that their intervention had a positive effect on adherence [25, 27–32, 34–36]. One of these studies [31] reported a positive effect on adherence to statins after 6 months, but no positive effect at 12 and 90 months. Another study [27] reported a positive effect on adherence only in a sub-group of women who had the electronic reminder in a secondary prevention setting. The other two studies [26, 33] reported no effect.

Because of the heterogeneity of the outcome measures (Table 2), pooled effects by intervention types were not estimated. However, Table 2 reports the presence or absence of effect on adherence for each study, along with its direction and magnitude.

### Results of syntheses

Interventions were categorized into five out of the eight possible categories previously mentioned.

*Simplification of drug regimen*: One study attempted to increase patient adherence to statin therapy by using a polypill [32] combining ezetimibe and statins. This approach reports a RR estimating the association of high adherence to treatment (PDC > 75%) (Table 2) and single-pill combination of statin and ezetimibe vs. two-pill or separate administration of the two drugs of 2.12 (95% CI: 1.89–2.38) in favor of the single-pill combination for patients aged 65 to 80 years old.

*Patient education and information*: Two studies [31, 33] attempted to increase adherence using education-based strategies. The results of Eussen et al. [33] were inconclusive regarding the effect of in-person counseling visits in a pharmacy setting on the incidence of discontinuation therapy in patients aged > 65 years old (HR = 0.903 [95% CI: 0.569–1.433]). However, Qvist et al. [31] reported a 10.1% (95% CI: 0.9–19.4) difference in the proportion of adherent (PDC ≥ 80%) males aged 65 to 74 years old in favor of telephone-based counseling at 6 months, but not at 12 or 60 months.

#### Intensified patient care

Four trials [25–27, 34] examined, with diverging results, whether intensified patient care could have a positive impact on statin adherence. For example, in the study by Casula et al. [34], an informative educational intervention aimed at general practitioners (which we interpretated as having a downstream intensification of patient care even though this was not specifically identified within the manuscript) succeeded in increasing the MPR of 65–79 years old patients by an absolute increment of 6.3%, and by 8.3% in patients who were aged ≥ 80 years old. The authors declared this increase to be significant, but no confidence interval or *p*-value was shown in the article. Similarly, Faridi et al. [25] showed that providing a first outpatient visit in the first week after discharge after a hospitalization for ST-elevation myocardial infarction or a non-ST-elevation myocardial infarction had a positive effect on patient adherence in patients when compared to first outpatient visits that took place more than 6 weeks after discharge. On the other hand, results by Guerard et al. [26] and Kooy et al. [27], using a comprehensive wellness assessment program or patient counselling combined with an electronic reminder device respectively, did not show any impact on statin adherence. Of note, although general results were inconclusive, Kooy et al. [27] did report that using only the electronic reminder device had a positive effect on patient adherence in women in a secondary prevention setting (adjusted OR = 8.26 [2.20–31.0], *p* = 0.002).

#### Administrative improvements

Three studies [28–30] examined whether administrative improvements could increase statin adherence in the target population. Ivers et al. [28] demonstrated an increase in patient adherence measured by the percentage of patients with a high PDC (> 80%) if patients were given a higher supply of statins in their initial prescription, with an adjusted OR of 2.0 (1.7–2.4) for a supply of 31–60 days and of 3.0 (2.6–3.4) for a supply of more than 60 days when compared to a supply of less than 31 days. Lester et al. [29] showed that patients enrolled in an automatic prescription refill program had higher adjusted odds of being adherent (PDC > 80%) compared to patients who received usual care (adjusted OR = 1.51 [1.26–1.82]). Schmittdiel et al. [30] studied the impact of increasing mean days supply of statins, higher use of mail-order pharmacies, a lower price of generic drug co-payments and a lower annual out-of-pocket maximum on statin adherence based on observations made in various settings. All these interventions showed a positive association with patient adherence to statin therapy, with adjusted odds ratios ranging from 1.02 to 1.61 in favor of the interventions.

#### Large-scale pharmacy-led automated telephone intervention

Two studies [35, 36] examined the impact of large-scale pharmacy-led automated telephone interventions on statin adherence. More specifically, Derose et al. [36] reported that providing educational information and an encouraging prompt to patients who recently received a statin prescription had a positive effect on the proportion of statin dispensation in individuals over 70 years old (OR = 2.32 [1.70–3.18]). Similarly, results from Vollmer et al. [35] showed that an interactive voice-recognition calls with or without personalized reminder letters in patients aged 71 years or older that were due or overdue for a statin refill also had a positive effect on patient adherence. More specifically, they showed that combining the call with a personalized letter showed a greater increase in PDC (+ 3.5% [1.2-5.8%]) than without (+ 2.9% [0.6-5.1%]) when compared to usual care [35].

## Discussion

In the present review, we identified five RTCs [27, 31, 33, 35, 36], five cohort studies [25, 26, 28, 30, 32] and two quasi-experimental studies [29, 34] published between 2010 and 2021 reporting on interventions attempting to increase statin adherence in older adults. Overall, our review suggests that simplifying patients’ drug regimen, administrative improvements and large-scale pharmacy-led automated telephone interventions tend to be effective when trying to improve older patients’ adherence to statins.

To the best of our knowledge, this review is the first to specifically examine interventions aimed at improving statin adherence amongst older adults. This is particularly important as the effectiveness of interventions aiming to improve statin adherence within this patient subset, for multiple reasons (e.g., administrative requirements, individuals’ financial capacity, complex drug regimens), could differ from what could be observed within an unrestricted adult population. Prior to conducting our review, two groups conducted two distinct systematic reviews of RCT interventions aimed at improving statin adherence within adult patients (no restriction in regards to patients’ age) [16, 37]. Some key differences in terms of results between our and their reviews warrant further discussion. For example, in the review by Schedlbauer et al. [37], interventions focusing on patient re-inforcement and reminders tended to have the best overall effect on adherence (four out of six selected studies showed improvements in patient adherence with average results ranging from 8 to 24%). However, none of the studies included in our review examined an intervention of this type; questions remain as to why this is the case. One potential explanation could be that by offering the intervention to an unrestricted adult population, investigators are maximizing its potential to improve adherence within a larger group of individuals. Another potential explanation could be that investigators designing such interventions could have a priori hypotheses (justified or not) that their intervention could be less effective within older patients. Regardless of the reason, considering their positive effect in the general adult population, their effect within older adults should be studied. This reasoning is further strengthened by comparing our results to those highlighted by Rash et al. [16]. Indeed, in their review, simplifying patients’ drug regimen had the greatest effect on patients’ adherence (with average results of three selected studies showing between 10% and 23% absolute increases in patients’ adherence). Though less common in the older adult population, we did identify one study that examined the impact of simplifying patients’ drug regimen on patients’ statin adherence [32]; their results highly supported this treatment strategy within older adults (Table 2). Although we cannot infer that this single study’s results can be reproduced within all older adult statin users, alignment of these results with those obtained by Rea et al. [32] warrant reproducing similar work within other jurisdictions.

Beyond these important results, our review also highlights the risk that, even amongst older adults, interventions’ effective profile may not be homogeneous. For example, though the intervention examined by Kooy et al. [27] failed to show a statistically significant increase in patient adherence within all patients, they showed a statistically significant increase in adherence among women hinting that individuals’ gender could influence treatment effect. Unfortunately, subgroup analyses were uncommon within the selected studies and no other group highlighted the presence of differential results based on individuals’ gender. That being said, just as interventions’ effectiveness can differ in function of individuals’ age and gender, they could differ in function of other key characteristics as well. Future work in this area needs to better acknowledge that older patients can represent a heterogeneous group and favor prespecified subgroup analyses to examine for presence of differential subgroup effects.

The evidence included in this review has limitations. Most studies reporting on interventions aiming to increase statin adherence in patients do not specifically target older adults. Indeed, only six out of 12 included studies [25–30] focused on this population. While some studies stratify analyses by age group, these analyses are based on a lower number of patients, which can lead to a loss of statistical power that might impact the ability to detect intervention effects. Beyond this fact, our assessment of the risk of bias within the selected studies identified also raised several issues (Additional File 4). Though issues differed between studies and study types, patient attrition was a common problem as only three [25, 27, 30] out of the 12 selected studies reported on it. Moreover, most studies that did not report attrition also failed to report on possible reasons for attrition that would help the reviewers make informed decisions about the degree of bias this introduces. Attrition in these cases could be particularly problematic as it could easily be explained by individuals refusing the interventions or requirements of. If that were the case, the true effect of the interventions we reviewed could be poorer than reported. Similarly, our review also highlights that patients’ long-term adherence to statin is lacking. Indeed, though all 12 studies included at least some individuals followed up to at least 12 months (proportion of individuals followed up to 12 months differing in function of study design), only three [26, 28, 31] of these allowed the follow-up to extend beyond 12 months. When focusing specifically on these three studies, only one [28] showed a significant positive effect after 12 months (investigators assessing patients’ adherence at 18 months). The other two did not show a significant effect and were in intervention categories that yielded mixed results in older adults (intensified patient care) and in both older adults and the general population (patient education and information) [16, 37]. Unfortunately, such results do not allow us to fully define if and how the effectiveness profiles of these interventions vary over time. On one hand, it is possible that adherence-enhancing interventions might have shown a greater benefit if follow-ups had been longer as statin adherence tends to fall substantially over time. On the other, greater follow-up could also have shown that their effectiveness plateaus or even declines beyond that 1-year mark. Lastly, all 12 included studies were performed in high-income countries, which limits the generalisability of the results for low to middle-income countries.

The synthesis procedure used in this review has limitations as well. Even if interventions are pragmatically regrouped into categories, substantial differences remain in the nature of interventions within the same category. Because the studies differed in terms of designs, outcome measures (RR/OR/HR/%), study populations (including in terms of indication for statin therapy) and time frames, we could not calculate pooled effects and only reported on the direction and statistical significance of the effect of included interventions. As such, we only conducted a qualitative, narrative synthesis of results. All findings are thus subject to the limitations of this approach. Furthermore, though our review identifies some interventions that succeeded and others that fail to improve patient adherence, we were unable to fully explain why this was the case. Unfortunately, out of the 12 studies included in our review, only Qvist et al. [31] specifically examined why participants in their trial did not adhere to their treatment. Another limitation of the used intervention categories is that they do not classify interventions by the reasons for patient nonadherence. Patient adherence is a complex issue and taking patients’ reasons for nonadherence into account when devising an intervention or a way to classify them could lead to more comparability and better outcomes. This issue is further complexified by the fact that some interventions (e.g., automated refills) could bias some of our commonly use adherence measures, such as PDC especially if based on administrative data. This was indeed the case in three of our studies [28–30]. Although all three manuscripts recognized that drug possession does not guarantee drug consumption, the retrospective nature of these studies limits the feasibility of directly confirming consumption with those included in their study.

## Conclusions

In conclusion, the evidence suggests that simplifying patients’ drug regimen, administrative improvements, and large-scale pharmacy-led automated telephone interventions may have positive effects on patient adherence to statin therapy, while education-based strategies and intensified patient care had mixed results. Although our review was restricted to older patients, we noticed that most studies tended to apply their intervention to the general adult population. As a result, important gaps in knowledge remain regarding interventions to improve statin adherence specifically in older adults. Moreover, patient adherence can be influenced by different factors such as medication side effects or fear of side effects, patient beliefs and memory [38, 39]. Tailoring interventions to address patient’s reasons for nonadherence in this vulnerable population and better understanding the mechanisms underlying adherence might lead to strategies that are more effective in improving statin adherence [38].

### Electronic supplementary material

Below is the link to the electronic supplementary material.

Supplementary Material 1

Supplementary Material 2

Supplementary Material 3

Supplementary Material 4

## Data Availability

The datasets used and/or analysed during the current study are available from the corresponding author on reasonable request.

## Abbreviations

CVD: Cardiovascular disease
RCT: Randomized clinical trial
PDC: Percentage of days covered
MPR: Medication possession ratio
NR: Not reported
AAA: Abdominal Aortic Aneurysm
PAD: Peripheral Artery Disease
STEMI: ST-elevation myocardial infarction
NSTEMI: Non-ST-elevation myocardial infarction
CWA: Comprehensive Wellness Assessment
CAD: Coronary Artery Disease
ACEI: Angiotensin-converting enzyme inhibitors
ARB: Angiotensin II receptor blockers
IVR: Interactive voice recognition
IVR +: Interactive voice recognition with a personalized reminder letter
RR: Risk Ratio
CI: Confidence Interval
PD: Proportion Difference
HR: Hazard Ratio
OR: Odds Ratio
IRR: Incidence rate ratio
UC: Usual Care
NHS: National Health Service
y.o.: Years old
