# Supplementary material for: Strategies aiming to improve statin therapy adherence in older adults: a systematic review
Source: BMC Geriatr. 2024 May 21;24:444. doi: 10.1186/s12877-024-05031-z (PMC11110402; doi:10.1186/s12877-024-05031-z)
Supplement: Supplementary file 2 — Supplementary Material 2 [file 12877_2024_5031_MOESM2_ESM.docx]

**Additional File 2: Research Strategies**

| PubMed | |
| --- | --- |
| Outcome (adherence) | *"Medication Adherence" [Mesh] OR "Treatment Refusal" [Mesh] OR "Patient dropouts" [Mesh] OR "Medication Adherence" [tiab] OR "Medication Nonadherence" [tiab] OR "Medication Noncompliance" [tiab] OR "Medication Non-Adherence" [tiab] OR "Medication Non Adherence" [tiab] OR "Medication Persistence" [tiab] OR "Medication Compliance" [tiab] OR "Medication Non-Compliance" [tiab] OR "Medication Non Compliance" [tiab] OR "Pharmaceutical treatment" [tiab] OR "Drug regimen" [tiab] OR "regimen adherence" [tiab] OR "Patient compliance" [tiab] OR "Patient adherence" [tiab] OR "Patient non-compliance" [tiab] OR "Patient noncompliance" [tiab] OR "Patient nonadherence" [tiab] OR "Adherence to medication treatment" [tiab] OR "medication prescribed" [tiab] OR "medication taking" [tiab] OR "Patient Dropout" [tiab] OR "Refusal of Treatment" [tiab] OR "Patient Refusal of Treatment" [tiab] OR "drug adherence" [tiab] OR "drug compliance" [tiab] OR "medication intake" [tiab] OR "drug intake" [tiab] OR "drug regimen" [tiab] OR "therapy adherence" [tiab] OR "therapy compliance" [tiab] OR "adherence to therapy" [tiab] OR "adherence to treatment" [tiab]* |
| Medication (statin) | *"Hydroxymethylglutaryl-CoA Reductase Inhibitors" [Mesh] OR "Hydroxymethylglutaryl-CoA Reductase Inhibitors" [Pharmacological Action] OR "HMG-CoA Reductase Inhibitors" [tiab] OR "HMG CoA Reductase Inhibitors" [tiab] OR "HMG-CoA Statins" [tiab] OR "Hydroxymethylglutaryl-CoA Inhibitors" [tiab] OR Statins[tiab] OR Statin[tiab] OR "Hydroxymethylglutaryl-Coenzyme A Inhibitors" [tiab] OR Lovastatin[tiab] OR Atorvastatin[tiab] OR Cerivastatin[tiab] OR Fluvastatin[tiab] OR Rosuvastatin[tiab] OR Simvastatin[tiab] OR Pravastatin[tiab] OR Pitavastatin[tiab] OR "Lipid Lowering" [tiab] OR "Lipid-lowering" [tiab]OR Lipitor[tiab] OR Lescol[tiab] OR Mevacor[tiab] OR Pravachol[tiab] OR Crestor[tiab] OR Zocor[tiab]* |
| Population (aged 65 years and older) | *Aged [Mesh] OR "Aged" [Mesh]* *OR Aged[tiab] OR geriatric*[tiab] OR elder*[tiab] OR senior*[tiab] OR ((old[tiab] OR oldest*[tiab] OR older*[tiab]) AND (person[tiab] OR adult*[tiab] OR individual*[tiab] OR people[tiab] OR patient[tiab]))* |
| Embase | |
| Outcome (adherence) | *'medication compliance'/exp OR 'treatment refusal'/exp OR 'patient dropout'/exp OR 'Medication Adherence':ab,ti OR 'Medication Nonadherence':ab,ti OR 'Medication Noncompliance':ab,ti OR 'Medication Non-Adherence':ab,ti OR 'Medication Non Adherence':ab,ti OR 'Medication Persistence':ab,ti OR 'Medication Compliance':ab,ti OR 'Medication Non-Compliance':ab,ti OR 'Medication Non Compliance':ab,ti OR 'Pharmaceutical treatment':ab,ti OR 'Drug regimen':ab,ti OR 'regimen adherence':ab,ti OR 'Patient compliance':ab,ti OR 'Patient adherence':ab,ti OR 'Patient non-compliance':ab,ti OR 'Patient noncompliance':ab,ti OR 'Patient nonadherence':ab,ti OR 'Adherence to medication treatment':ab,ti OR 'medication prescribed':ab,ti OR 'medication taking':ab,ti OR 'Patient Dropout':ab,ti OR 'Refusal of Treatment':ab,ti OR 'Patient Refusal of Treatment':ab,ti OR 'drug adherence':ab,ti OR 'drug compliance':ab,ti OR 'medication intake':ab,ti OR 'drug intake':ab,ti OR 'drug regimen':ab,ti OR 'therapy adherence':ab,ti OR 'therapy compliance':ab,ti OR 'adherence to therapy':ab,ti OR 'adherence to treatment':ab,ti* |
| Medication (statin) | *'hydroxymethylglutaryl coenzyme A reductase inhibitor'/exp OR 'HMG-CoA Reductase Inhibitors':ab,ti OR 'HMG CoA Reductase Inhibitors':ab,ti OR 'HMG-CoA Statins':ab,ti OR 'Hydroxymethylglutaryl-CoA Inhibitors':ab,ti OR 'Statins':ab,ti OR 'Statin':ab,ti OR 'Hydroxymethylglutaryl-Coenzyme A Inhibitors':ab,ti OR 'Lovastatin':ab,ti OR 'Atorvastatin':ab,ti OR 'Cerivastatin':ab,ti OR 'Fluvastatin':ab,ti OR 'Rosuvastatin':ab,ti OR 'Simvastatin':ab,ti OR 'Pravastatin':ab,ti OR 'Pitavastatin':ab,ti OR 'Lipid-Lowering':ab,ti OR 'Lipid-lowering':ab,ti OR 'Lipitor':ab,ti OR 'Lescol':ab,ti OR 'Mevacor':ab,ti OR 'Pravachol':ab,ti OR 'Crestor':ab,ti OR 'Zocor':ab,ti* |
| Population (aged 65 years and older) | *'aged'/exp OR Aged:ab,ti OR geriatric*:ab,ti OR elder*:ab,ti OR senior*:ab,ti OR ((old:ab,ti OR oldest*:ab,ti OR older*:ab,ti) AND (person:ab,ti OR adult*:ab,ti OR individual*:ab,ti OR people:ab,ti OR patient:ab,ti)) OR 'Very Elderly':ab,ti OR 'Oldest Old':ab,ti OR 'Nonagenarian*':ab,ti OR 'Octogenarian*':ab,ti OR 'Centenarian*':ab,ti* |
| PsycInfo | |
| Outcome (adherence) | *"Treatment Refusal"/ OR "Treatment Refusal".ti,ab,id OR "Treatment dropouts"/ OR "Treatment dropouts".ti,ab,id OR "medication compliance"/ OR "medication compliance".ti,ab,id OR "treatment refusal"/ OR "treatment refusal".ti,ab,id OR "patient dropout"/ OR "patient dropout".ti,ab,id OR "Medication Adherence".ti,ab,id OR "Medication Nonadherence".ti,ab,id OR "Medication Noncompliance".ti,ab,id OR "Medication Non-Adherence".ti,ab,id OR "Medication Non Adherence".ti,ab,id OR "Medication Persistence".ti,ab,id OR "Medication Compliance".ti,ab,id OR "Medication Non-Compliance".ti,ab,id OR "Medication Non Compliance".ti,ab,id OR "Pharmaceutical treatment".ti,ab,id OR "Drug regimen".ti,ab,id OR "regimen adherence".ti,ab,id OR "Patient compliance".ti,ab,id OR "Patient adherence".ti,ab,id OR "Patient non-compliance".ti,ab,id OR "Patient noncompliance".ti,ab,id OR "Patient nonadherence".ti,ab,id OR "Adherence to medication treatment".ti,ab,id OR "medication prescribed".ti,ab,id OR "medication taking".ti,ab,id OR "Patient Dropout*".ti,ab,id OR "Refusal of Treatment".ti,ab,id OR "Patient Refusal of Treatment".ti,ab,id OR "Treatment refusal".ti,ab,id OR "drug adherence".ti,ab,id OR "drug compliance".ti,ab,id OR "medication intake".ti,ab,id OR "drug intake".ti,ab,id OR "drug regimen".ti,ab,id OR "therapy adherence".ti,ab,id OR "therapy compliance".ti,ab,id OR "adherence to therapy".ti,ab,id OR "adherence to treatment".ti,ab,id* |
| Medication (statin) | *Statins/ OR Statins.ti,ab,id OR "HMG-CoA Reductase Inhibitors".ti,ab,id OR "HMG CoA Reductase Inhibitors".ti,ab,id OR "HMG-CoA Statins".ti,ab,id OR "Hydroxymethylglutaryl-CoA Inhibitors".ti,ab,id OR Statin.ti,ab,id OR "Hydroxymethylglutaryl-Coenzyme A Inhibitor*".ti,ab,id OR Lovastatin.ti,ab,id OR Atorvastatin.ti,ab,id OR Cerivastatin.ti,ab,id OR Fluvastatin.ti,ab,id OR Rosuvastatin.ti,ab,id OR Simvastatin.ti,ab,id OR Pravastatin.ti,ab,id OR Pitavastatin.ti,ab,id OR "Lipid-Lowering".ti,ab,id OR "Lipid-lowering".ti,ab,id OR Lipitor.ti,ab,id OR Lescol.ti,ab,id OR Mevacor.ti,ab,id OR Pravachol.ti,ab,id OR Crestor.ti,ab,id OR Zocor.ti,ab,id* |
| Population (aged 65 years and older) | *Aged/ OR "Aged".ti,ab,id OR Elderly/ OR "Elderly".ti,ab,id OR "Very Elderly".ti,ab,id OR "Oldest Old".ti,ab,id OR "Nonagenarian*".ti,ab,id OR "Octogenarian*".ti,ab,id OR "Centenarian*".ti,ab,id OR geriatric*.ti,ab,id OR elder*.ti,ab,id OR senior*.ti,ab,id OR ((old.ti,ab,id OR oldest*.ti,ab,id OR older*.ti,ab,id) AND (person.ti,ab,id OR adult.ti,ab,id OR individual*.ti,ab,id OR people.ti,ab,id OR patient.ti,ab,id))* |
| Web of Science | |
| Outcome (adherence) | *I=("Treatment Refusal" OR "Treatment dropouts" OR "Medication Adherence" OR "Medication Nonadherence" OR "Medication Noncompliance" OR "Medication Non-Adherence" OR "Medication Non Adherence" OR "Medication Persistence" OR "Medication Compliance" OR "Medication Non-Compliance" OR "Medication Non Compliance" OR "Pharmaceutical treatment" OR "Drug regimen" OR "regimen adherence" OR "Patient compliance" OR "Patient adherence" OR "Patient non-compliance" OR "Patient noncompliance" OR "Patient nonadherence" OR "Adherence to medication treatment" OR "medication prescribed" OR "medication taking" OR "Patient Dropout*" OR "Refusal of Treatment" OR "Patient Refusal of Treatment" OR "Treatment refusal" OR "drug adherence" OR "drug compliance" OR "medication intake" OR "drug intake" OR "drug regimen" OR "therapy adherence" OR "therapy compliance" OR "adherence to therapy" OR "adherence to treatment") OR TS=("Treatment Refusal" OR "Treatment dropouts" OR "Medication Adherence" OR "Medication Nonadherence" OR "Medication Noncompliance" OR "Medication Non-Adherence" OR "Medication Non Adherence" OR "Medication Persistence" OR "Medication Compliance" OR "Medication Non-Compliance" OR "Medication Non Compliance" OR "Pharmaceutical treatment" OR "Drug regimen" OR "regimen adherence" OR "Patient compliance" OR "Patient adherence" OR "Patient non-compliance" OR "Patient noncompliance" OR "Patient nonadherence" OR "Adherence to medication treatment" OR "medication prescribed" OR "medication taking" OR "Patient Dropout*" OR "Refusal of Treatment" OR "Patient Refusal of Treatment" OR "Treatment refusal" OR "drug adherence" OR "drug compliance" OR "medication intake" OR "drug intake" OR "drug regimen" OR "therapy adherence" OR "therapy compliance" OR "adherence to therapy" OR "adherence to treatment")* |
| Medication (statin) | *TI= (Statins OR "HMG-CoA Reductase Inhibitors" OR "HMG CoA Reductase Inhibitors" OR "HMG-CoA Statins" OR "Hydroxymethylglutaryl-CoA Inhibitors" OR Statin.ti,ab,id OR "Hydroxymethylglutaryl-Coenzyme A Inhibitor*" OR Lovastatin OR Atorvastatin OR Cerivastatin OR Fluvastatin OR Rosuvastatin OR Simvastatin OR Pravastatin OR Pitavastatin OR "Lipid-Lowering" OR "Lipid-lowering" OR Lipitor OR Lescol OR Mevacor OR Pravachol OR Crestor OR Zocor) OR TS= (Statins OR "HMG-CoA Reductase Inhibitors" OR "HMG CoA Reductase Inhibitors" OR "HMG-CoA Statins" OR "Hydroxymethylglutaryl-CoA Inhibitors" OR Statin.ti,ab,id OR "Hydroxymethylglutaryl-Coenzyme A Inhibitor*" OR Lovastatin OR Atorvastatin OR Cerivastatin OR Fluvastatin OR Rosuvastatin OR Simvastatin OR Pravastatin OR Pitavastatin OR "Lipid-Lowering" OR "Lipid-lowering" OR Lipitor OR Lescol OR Mevacor OR Pravachol OR Crestor OR Zocor)* |
| Population (aged 65 years and older) | *TI= (Aged OR Elderly OR "Very Elderly" OR "Oldest Old" OR "Nonagenarian*" OR "Octogenarian*" OR "Centenarian*" OR geriatric* OR elder* OR senior* OR ((old OR oldest* OR older*) AND (person OR adult OR individual* OR people OR patient))) OR TS= (Aged OR Elderly OR "Very Elderly" OR "Oldest Old" OR "Nonagenarian*" OR "Octogenarian*" OR "Centenarian*" OR geriatric* OR elder* OR senior* OR ((old OR oldest* OR older*) AND (person OR adult OR individual* OR people OR patient)))* |
| CINAHL | |
| Outcome (adherence) | *MH "Medication Compliance" OR MH "Treatment Refusal" OR MH "Patient dropouts" OR Ti "Medication Adherence" OR Ti "Medication Nonadherence" OR Ti "Medication Noncompliance" OR Ti "Medication Non-Adherence" OR Ti "Medication Non Adherence" OR Ti "Medication Persistence" OR Ti "Medication Compliance" OR Ti "Medication Non-Compliance" OR Ti "Medication Non Compliance" OR Ti "Pharmaceutical treatment" OR Ti "Drug regimen" OR Ti "regimen adherence" OR Ti "Patient compliance" OR Ti "Patient adherence" OR Ti "Patient non-compliance" OR Ti "Patient noncompliance" OR Ti "Patient nonadherence" OR Ti "Adherence to medication treatment" OR Ti "medication prescribed" OR Ti "medication taking" OR Ti "Patient Dropout*" OR Ti "Refusal of Treatment" OR Ti "Patient Refusal of Treatment" OR Ti "drug adherence" OR Ti "drug compliance" OR Ti "medication intake" OR Ti "drug intake" OR Ti "drug regimen" OR Ti "therapy adherence" OR Ti "therapy compliance" OR Ti "adherence to therapy" OR Ti "adherence to treatment" OR Ab "Medication Adherence" OR Ab "Medication Nonadherence" OR Ab "Medication Noncompliance" OR Ab "Medication Non-Adherence" OR Ab "Medication Non Adherence" OR Ab "Medication Persistence" OR Ab "Medication Compliance" OR Ab "Medication Non-Compliance" OR Ab "Medication Non Compliance" OR Ab "Pharmaceutical treatment" OR Ab "Drug regimen" OR Ab "regimen adherence" OR Ab "Patient compliance" OR Ab "Patient adherence" OR Ab "Patient non-compliance" OR Ab "Patient noncompliance" OR Ab "Patient nonadherence" OR Ab "Adherence to medication treatment" OR Ab "medication prescribed" OR Ab "medication taking" OR Ab "Patient Dropout*" OR Ab "Refusal of Treatment" OR Ab "Patient Refusal of Treatment" OR Ab "drug adherence" OR Ab "drug compliance" OR Ab "medication intake" OR Ab "drug intake" OR Ab "drug regimen" OR Ab "therapy adherence" OR Ab "therapy compliance" OR Ab "adherence to therapy" OR Ab "adherence to treatment"* |
| Medication (statin) | *MH Statins OR Ti "HMG-CoA Reductase Inhibitors" OR Ti "HMG CoA Reductase Inhibitors" OR Ti "HMG-CoA Statins" OR Ti "Hydroxymethylglutaryl-CoA Inhibitors" OR Ti "Hydroxymethylglutaryl-CoA Reductase Inhibitors" OR Ti "Hydroxymethylglutaryl-Coenzyme A Inhibitors" OR Ti Lovastatin OR Ti Atorvastatin OR Ti Cerivastatin OR Ti Fluvastatin OR Ti Rosuvastatin OR Ti Simvastatin OR Ti Pravastatin OR Ti Pitavastatin OR Ti "Lipid-Lowering" OR Ti "Lipid-lowering" OR Ti Lipitor OR Ti Lescol OR Ti Mevacor OR Ti Pravachol OR Ti Crestor OR Ti Zocor OR Ab "HMG-CoA Reductase Inhibitors" OR Ab "HMG CoA Reductase Inhibitors" OR Ab "HMG-CoA Statins" OR Ab "Hydroxymethylglutaryl-CoA Inhibitors" OR Ab "Hydroxymethylglutaryl-CoA Reductase Inhibitors" OR Ab "Hydroxymethylglutaryl-Coenzyme A Inhibitors" OR Ab Lovastatin OR Ab Atorvastatin OR Ab Cerivastatin OR Ab Fluvastatin OR Ab Rosuvastatin OR Ab Simvastatin OR Ab Pravastatin OR Ab Pitavastatin OR Ab "Lipid-Lowering" OR Ab "Lipid-lowering" OR Ab Lipitor OR Ab Lescol OR Ab Mevacor OR Ab Pravachol OR Ab Crestor OR Ab Zocor* |
| Population (aged 65 years and older) | *MH Aged OR Ti Aged OR Ti Elderly OR Ti geriatric* OR Ti elder* OR Ti senior* OR ((Ti old OR Ti oldest* OR Ti older*) AND (Ti person OR Ti adult* OR Ti individual* OR Ti people OR Ti patient)) OR Ti Nonagenarian* OR Ti Octogenarian* OR Ti Centenarian* OR Ab Aged OR Ab Elderly OR Ab geriatric* OR Ab elder* OR Ab senior* OR ((Ab old OR Ab oldest* OR Ab older*) AND (Ab person OR Ab adult* OR Ab individual* OR Ab people OR Ab patient)) OR Ab Nonagenarian* OR Ab Octogenarian* OR Ab Centenarian** |
