# Supplementary material for: Strategies aiming to improve statin therapy adherence in older adults: a systematic review
Source: BMC Geriatr. 2024 May 21;24:444. doi: 10.1186/s12877-024-05031-z (PMC11110402; doi:10.1186/s12877-024-05031-z)
Supplement: Supplementary file 3 — Supplementary Material 3 [file 12877_2024_5031_MOESM3_ESM.docx]

**Additional file 3: Intervention Characteristics**

Rea *et al.* (2021): The intervention, conducted with Healthcare utilization databases of Lombardy on adults aged 40-80 years old that are beneficiaries of the National Health Service and that were prescribed a statin during 2011-2013 consisted of comparing proportion of days covered and incidence of treatment discontinuation when comparing patients who receive statin and ezetimibe prescribed as a single pill formulation to patients who received them as separate pills with a follow-up of 12 months.

Eussen *et al.* (2010): The intervention, conducted in a community pharmacy setting in the Netherlands on new users of statins aged 18 years or older capable of visiting the pharmacy, consisted of comparing the incidence of statin treatment discontinuation between patients going to their pharmacy for 5 individual counseling sessions, each lasting 5-10 minutes compared to usual care with a follow-up period of 12 months.

Qvist *et al.* (2020): The intervention, conducted in Denmark on males aged between 65-74 years old in the screening arm of the VIVA trial diagnosed with abdominal aortic aneurysm or peripheral artery disease and non-users of statin or antithrombotic treatment at baseline, compared the proportions of days covered at 6, 12 and 60 months between patients who received a telephone-based interview and counselling performed by a study nurse, based on a pragmatically designed semi-structured questionnaire to usual care, with a maximum follow-up of 60 months.

Casula *et al.* (2015): The intervention, conducted in Lombardy on pre-intervention patients with the first statin prescription between 1 January 2007 and 30 June 2007 and on post-intervention patients with the first statin prescription between 1 July 2008 and 31 December 2008 compared the medication possession ratio between patients who visited general practitioners who received an informative and educational intervention on patient statin adherence and patients receiving usual care, with a follow-up of 12 months.

Faridi *et al.* (2016): The intervention, conducted in the US on patients discharged between January 2, 2007, and October 1, 2010 who are enrolled in Medicare Part D prescription coverage at least 90 days before discharge compared the proportion of days covered at 90 days and one year between patients receiving an earlier first outpatient visit after discharge for ST-elevation myocardial infarction or non-ST-elevation myocardial infarction and patients receiving usual care, with a follow-up of 3 & 12 months.

Guerard *et al.* (2018): The intervention, conducted in the US on Medicare Advantage members who received a new diagnosis of diabetes since 2010 compared the proportion of days covered between patients receiving a comprehensive wellness assessment in the preceding 12 months of the study and patients receiving usual care, with a follow-up of 60 months.

Kooy *et al.* (2013): The intervention was conducted in a community pharmacy setting in the Netherlands on patients who received a prescription for a statin in the preceding month, received a prescription for the same statin between 12 and 18 months prior to that prescription and have a refill adherence between 50 and 80% during the 365 days prior to the last statin prescription covered by the same statin. The authors compared the proportion of days covered between patients receiving a 10-minute pharmacist counseling about non-adherence and a compliance card that signals every 24 hours when compared to usual care. They also compared the use of only the compliance card compared to counseling and the card, and only the compliance card when compared to usual care. The follow-up of the study was of 12 months.

Ivers *et al.* (2016): The intervention was conducted in a pharmacy setting on patients aged ≥65 years on or before January 1, 2014 taking at least 1 medication in the therapeutic categories used in calculating the proportion of days covered measures, at least 2 prescription refills of the included medications during the 2014 calendar year and a first fill for included medications occurring at least 91 days before the end of the study period. The authors compared the proportion of days covered between patients who received an increase in the number of days supplied in the initial prescription fill for each medication class when compared to usual care, with a follow-up of 18 months.

Schmittdiel *et al.* (2015): This article tested multiple interventions conducted in the US on patients aged ≥65 years as of January 1, 2010 having diabetes in 2010 (at least 2 outpatient diabetes ICD-9 diagnosis codes within a 2-year window since the start of 2000). The authors compared the proportion of days covered between patients receiving one of these interventions: mean days supply of drugs in therapeutic category in 2010, percentage of drugs in therapeutic category refilled through mail order pharmacy in 2010, generic drug copayment for 30-day supply in 2010, annual individual out-of-pocket maximum in January 2010 compared to usual care. The follow-up for these interventions was 12 months.

Derose *et al.* (2013): The intervention was conducted in a health plan pharmacy setting on patients with a prescription for a statin or combination drug containing a statin, no record of such a drug dispensed within 365 days before the index prescription rate, 1 or more years of membership from prescription date, no gap in enrollment more than 30 days during the past year and no record of the statin prescription being filled at a health plan pharmacy after 1 to 2 weeks. The authors compared the proportion of dispensation between patients who were provided with educational information and an encouraging prompt to adhere to a recently prescribed statin and patients who received usual care, with a follow-up of 32 to 39 days.

Vollmer *et al.* (2014): The intervention was conducted in the US on patients aged ≥40 years with documented diabetes or cardiovascular disease at time of randomization and having at least one dispensing of an angiotensin-converting enzyme inhibitors (ACEI), angiotensin II receptor blockers (ARB), or statin in the preceding 12 months, suboptimal adherence (medication possession ratio <0.9) to either statins or ACEI/ARBs in the preceding 12 months, having a continuous health plan membership for the 12 months prior to randomization and qualifying for an intervention call at the time of randomization. The authors compared the proportion of days covered between patients who received interactive voice recognition calls with or without a personalized reminder letter if patients were due or overdue for a statin refill to patients who received usual care. The follow-up time was of 12 months.
