# Supplementary material for: Strategies aiming to improve statin therapy adherence in older adults: a systematic review
Source: BMC Geriatr. 2024 May 21;24:444. doi: 10.1186/s12877-024-05031-z (PMC11110402; doi:10.1186/s12877-024-05031-z)
Supplement: Supplementary file 4 — Supplementary Material 4 [file 12877_2024_5031_MOESM4_ESM.docx]

**Additional file 4: Risk of Bias, filled Joanna Briggs Institute Forms**

**Additional file 4a: Risk of Bias in Randomized Controlled Trials**

| Article | Derose et al. | | Eussen et al. | Kooy et al. | Qvist et al. | Vollmer et al. |
| --- | --- | --- | --- | --- | --- | --- |
| Questions |  |  | |  |  |  |
| 1. Was true randomization used for assignment of participants to treatment groups? | Yes | | Yes | Yes | Yes | Yes |
| 2.  Was allocation to treatment groups concealed? | Yes | | Yes | Yes | Yes | Yes |
| 3.  Were treatment groups similar at the baseline? | Unclear | | No | Yes | Yes | Unclear |
| 4.  Were participants blind to treatment assignment? | NA | | NA | NA | No | No |
| 5.  Were those delivering treatment blind to treatment assignment? | NA | | NA | No | No | No |
| 6. Were outcomes assessors blind to treatment assignment? | Yes | | Yes | Unclear | No | Unclear |
| 7. Were treatment groups treated identically other than the intervention of interest? | Yes | | Yes | Yes | Yes | Yes |
| 8.  Was follow up complete and if not, were differences between groups in terms of their follow up adequately described and analyzed? | No | | No | No | Yes | Unclear |
| 9. Were participants analyzed in the groups to which they were randomized? | Yes | | Yes | Yes | Yes | Yes |
| 10. Were outcomes measured in the same way for treatment groups? | Yes | | Yes | Yes | Yes | Yes |
| 11. Were outcomes measured in a reliable way? | Unclear | | Unclear | Yes | Yes | Yes |
| 12. Was appropriate statistical analysis used? | Yes | | Yes | Yes | Yes | Yes |
| 13. Was the trial design appropriate and any deviations from the standard RCT design (individual randomization, parallel groups) accounted for in the conduct and analysis of the trial? | Yes | | Yes | Yes | Yes | Yes |
| Overall appraisal | Include | | Include | Include | Include | Include |

NA=Not applicable

**Additional file 4b: Risk of Bias in Cohort Studies**

| Article | Faridi et al. | Guerard et al. | Ivers et al. | Rea et al. | Schmittdiel et al. |
| --- | --- | --- | --- | --- | --- |
| Questions |  |  |  |  |  |
| 1. Were the two groups similar and recruited from the same population? | Yes | Unclear | Yes | Yes | Yes |
| 2. Were the exposures measured similarly to assign people to both exposed and unexposed groups? | Yes | Yes | Yes | Yes | Yes |
| 3. Was the exposure measured in a valid and reliable way? | Yes | Yes | Yes | Yes | Yes |
| 4. Were confounding factors identified? | Yes | Yes | Yes | Yes | Yes |
| 5. Were strategies to deal with confounding factors stated? | Yes | Yes | Yes | Yes | Yes |
| 6. Were the groups/participants free of the outcome at the start of the study (or at the moment of exposure)? | Yes | Yes | Unclear | Yes | Unclear |
| 7. Were the outcomes measured in a valid and reliable way? | Yes | Yes | Yes | Yes | Yes |
| 8. Was the follow up time reported and sufficient to be long enough for outcomes to occur? | Yes | Yes | Yes | Yes | Yes |
| 9. Was follow up complete, and if not, were the reasons to loss to follow up described and explored? | No | No | Yes | Yes | Unclear |
| 10. Were strategies to address incomplete follow up utilized? | No | No | NA | Yes | Unclear |
| 11. Was appropriate statistical analysis used? | Yes | Yes | Yes | Yes | Yes |
| Overall appraisal | Include | Include | Include | Include | Include |

NA=Not applicable

**Additional file 4c: Risk of Bias in Quasi-Experimental Studies**

| Article | Casula et al. | Lester et al. |
| --- | --- | --- |
| Questions |  |  |
| 1.  Is it clear in the study what is the ‘cause’ and what is the ‘effect’ (i.e. there is no confusion about which variable comes first)? | Yes | Yes |
| 2.  Were the participants included in any comparisons similar? | Unclear | No |
| 3.  Were the participants included in any comparisons receiving similar treatment/care, other than the exposure or intervention of interest? | Yes | Yes |
| 4. Was there a control group? | Yes | Yes |
| 5. Were there multiple measurements of the outcome both pre and post the intervention/exposure? | No | No |
| 6. Was follow up complete and if not, were differences between groups in terms of their follow up adequately described and analyzed? | Unclear | Unclear |
| 7. Were the outcomes of participants included in any comparisons measured in the same way? | Yes | Yes |
| 8. Were outcomes measured in a reliable way? | Yes | Yes |
| 9. Was appropriate statistical analysis used? | Yes | Yes |
| Overall appraisal | Include | Include |
